# Sacral neuromodulation: Therapy evolution

**DOI:** 10.4103/0970-1591.70576

**Published:** 2010

**Authors:** Jannah H. Thompson, Suzette E. Sutherland, Steven W. Siegel

**Affiliations:** Metropolitan Urology, Cornerstone Medical Specialty, 6025 Lake Road, Suite 100, Woodbury, MN, USA

**Keywords:** Refractory overactive, non-obstructive, neuromodulation

## Abstract

**Objectives:**

Sacral neuromodulation has gained increased worldwide acceptance as the standard of care in patients with refractory overactive bladder (OAB) and non-obstructive urinary retention (NOUR). This review will detail the evolution of the technology.

**Materials and Methods:**

The mechanism of action and advances in treatment, including tined lead, fluoroscopic imaging, and smaller implantable pulse generator (IPG) are reviewed. This discussion also explores expanding indications and future advances including interstitial cystitis, chronic pelvic pain, neurogenic bladder, fecal incontinence, constipation, and dysfunctional elimination syndrome in children.

**Results:**

Sacral neuromodulation (SNM) exerts its influence by modulation of sacral afferent inflow on storage and emptying reflexes. The tined lead allows for placement and stimulation to be performed in the outpatient setting under local anesthesia with mild sedation. Lead migration has been minimal and efficacy improved. The use of fluoroscopy has improved accuracy of lead placement and has led to renewed interest in bilateral percutaneous nerve evaluation (PNE). Bilateral PNE can be performed in the office setting under local anesthesia, making a trial of therapy less expensive and more attractive to patients. A smaller IPG has not only improved cosmesis, but decreased local discomfort and need for revision. The role for SNM continues to expand as clinical research identifies other applications for this therapy.

**Conclusions:**

Our understanding of SNM, as well as technological advances in therapy delivery, expands the pool of patients for which this form of therapy may prove beneficial. Less invasive instrumentation may even make this form of therapy appealing to patients without refractory symptoms.

## INTRODUCTION

Sacral neuromodulation (SNM) has been approved by the Food and Drug Administration (FDA) for the treatment of refractory voiding dysfunction since the late 1990s; urge incontinence (UI) since 1997, and urgency-frequency syndrome (U/F) and idiopathic, non-obstructive urinary retention (NOUR) since 1999.[1] This article reviews the evolution of this technology and the expanding indications for SNM therapy.

## BACKGROUND

Anticholinergic drugs have been the mainstay treatment for OAB in the United States for decades. While pharmacotherapy provides significant improvement in symptoms for many patients, the side effects (particularly dry mouth and constipation) lead many to discontinuation.[2] Although newer extended release formulations of tolterodine and oxybutynin have modestly improved the side effect profile, up to 50% of patients still withdraw from treatment after the first month.[3] Behavioral therapies such as dietary modifications, timed voiding, pelvic floor muscle biofeedback and physical therapy compliment or may replace pharmacotherapy as a first-line measure. SNM is a second-line alternative which addresses OAB by acting at the level of the primitive voiding reflex coordinating the bladder, sphincter and pelvic floor.

## HOW SACRAL NEUROMODULATION WORKS

**Figure 1:**
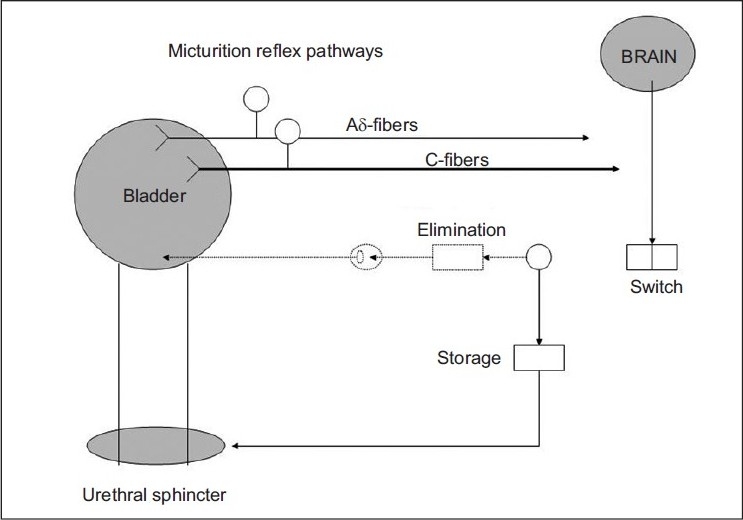
Micturition reflex pathways as outlined by deGroat. Note that the A-delta fibers and C-fibers provide afferent signaling to the brain as the bladder fills. The brain, in turn, signals spinal pathways, resulting in a “turning off” of the guarding reflex, relaxation of the external urethral sphincter, contraction of the detrusor, and voluntary voiding of urine

Schmidt and Tanagho’s pioneering work on dogs[4] and then humans lead us to a better understanding of the corresponding motor and sensory responses to sacral root stimulation that we use clinically during sacral lead placement [Table 1]. They discovered that stimulation-induced contraction of the urethral sphincter abolished detrusor contractions. The pudendal nerve plays a key role in bladder manipulation via the primitive voiding reflex.[5] Symptoms of incontinence or voiding dysfunction may represent an alteration of the pelvic neuromuscular environment by changes in the inhibitory and excitatory signals on the voiding reflex. SNM is likely to exert its influence by alteration of sacral afferent inflow on storage and emptying reflexes,[1] [Table 2]. If as a result of SNM, the neuromuscular guarding behavior is coordinated, then either voiding or storage may be facilitated. By decreasing the guarding reflex, voiding may be facilitated; by increasing it, appropriate storage may be promoted. The fact that the same treatment may be effective for symptoms of OAB and NOUR implies that the therapy is modulating the central nervous system at the level where switching between bladder emptying and storage occurs.[7] [Figure 1]

**Table 1:** Illustrates anticipated motor and sensory responses to stimulation at S2, S3 and S4

Level	Motor	Sensory
S2	Bellows (inward going of intergluteal fold), Clamp (A-P pinching of perineum/coccyx), dorsiflexion of foot, heel rotation, calf cramping	Genital
S3	Bellows, dorsiflexion of great toe, bottom of foot	Genital, perineal, anal
S4	Bellows	Anal

**Table 2:** Possible mechanisms of sacral nerve stimulation[1]

Inhibits postganglionic nerve terminals May inhibit primary afferents presynaptically May affects pudendal afferents that transmit somatic and visceral neurochemical signaling Inhibits spinal tract neurons involved in the micturition reflex May suppress indirectly guarding reflexes by turning off bladder afferent input to internal sphincter sympathetic or external urethral sphincter interneurons May activate bladder efferent to stimulate voiding while simultaneously “turning off” excitatory pathway to urethra

A study using positron emission tomography (PET) scanning of the brain during modulation of the sacral nerves identified several brain centers involved in proper urinary control cingulate cortex, midbrain and pons.[8] The therapeutic effect of SNM appeared to be associated with restoring brainstem auto-regulation. Afferent pathways as the main stimulation target is supported by evidence that the benefits of SNM occur at low electrical stimulation intensities, insufficient for activation of striated muscle movements. Inhibition of detrusor hyperreflexia can occur by inhibition of the bladder preganglionic neurons of the efferent limb of the micturition reflex and/or by direct inhibition of sacral interneuronal transmission in the afferent limb.[9]

## ADVANCES

Since European CE approval of Interstim™ Therapy (Medtronic Minneapolis, MN, USA) in 1994 and the North American FDA approval in October 1997, there have been continuous advances in technical and surgical aspects that have translated to improvement in use and efficacy. These include the tined permanent lead, routine use of fluoroscopy, and a smaller (IPG). Patient selection, preparation and technique for sacral lead placement and IPG implantation have been well described by Sutherland *et al*.[9] From an 11-year experience, 69%, 50%, and 35% of patients reported sustained subjective improvement as >50%, >80%, and >90%, respectively.[10] Good overall lead durability was reported at a mean of 22 months.

Medtronic conducted a post FDA-approval study to assess long-term efficacy and safety with patients followed for five years. In patients with urgency, statistically significant improvements were seen in number of leaks/day, number of heavy leaks/day and the number of pads/day. Excellent durability of this response to SNM therapy was also seen, with significant difference noted in the above mentioned parameters at every annual visit up to five years. At five years, the average number of leaks/day decreased from 9.6 to 3.9, with 58% of patients classified as clinically successful; the average number of heavy leaks/day decreased from 2.6 to 0.8, with 68% of patients classified as clinically successful; the average number of pads used/day decreased from 5.3 to 1.8, with 61% of patients classified as clinically successful.

In patients with urgency-frequency syndrome, average number of voids/day decreased significantly from 19.3 to 14.8, with a 40% clinical success rate at five years. Volume voided/void also improved from 92.3 ml to 165.2 ml at five years, with clinical success rate of 56% at five years. The clinical success rate in perceived degree of urgency was 56% at five years. An important finding in this study is the high correlation between the one- and five-year success rates for treated patients, indicating a good durability of response with SNM therapy. Of patients who were successfully treated at one-year follow-up, 84% with UI and 71% with UF continued to have a successful outcome at five years.[10]

## TINED LEAD

**Figure 2:**
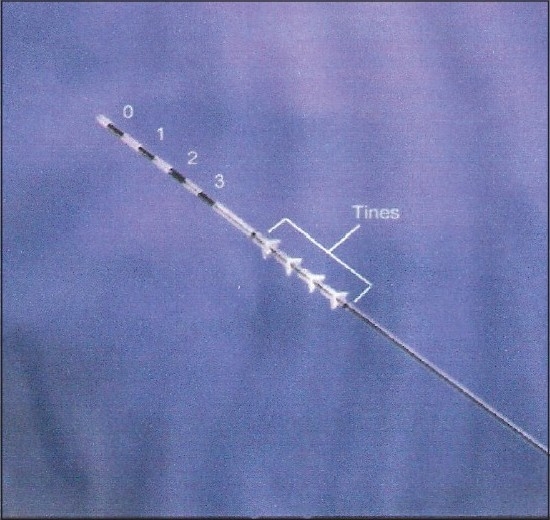
Tined lead. The series of four tines allows for anchoring to subcutaneous tissues. The lead also contains four electrodes labeled 0 to 3 to provide variation in the stimulation pattern.

Early permanent non-tined leads included bone- and fascial-anchored leads. Due to the invasive nature of bone-anchored leads general anesthesia was required, which eliminated the ability to utilize sensory information during intra-operative testing and placement. With the development of less invasive fascial-anchored leads, conscious sedation was now possible, and with that, the use of sensory information during intra-operative stimulation. With the advent of the percutaneous, self-anchoring tined lead (FDA-approved in 2002), the need for a large, deep incision was eliminated, allowing for the use of local anesthesia and mild conscious sedation[11] The patient’s sensory responses during intra-operative stimulation contribute greatly to proper lead placement in conjunction with motor responses and fluoroscopic clues. The use of intra-operative fluoroscopy has also proven beneficial for maximizing accurate lead placement. The quadripolar tined lead [Figure 2] consists of four sets of tines proximal to the electrodes, which engage the subcutaneous tissue and muscle around the lead to prevent migration and subsequent change in therapeutic efficacy.[7] Using the tined lead in a staged trial allows for a longer trial phase (two-three weeks) to assess improvements in voiding symptoms. Those with a successful staged trial may then proceed with the second stage implantation of the programmable IPG. Typically only one tined lead is placed during the staged procedure based on the side with the best motor and/or sensory responses at the time of the intra-operative testing. Stimulating only one side during the trial may limit the ability of the screening trial to fully assess symptomatic improvement. On the other hand, the permanent tined lead has four electrodes, allowing for a more precise placement along the course of the nerve. This increases the likelihood of accurate nerve stimulation and, with that, therapeutic programming options. Another advantage of the staged trial is a reduction in technical failures, since patients can be sure of the therapeutic benefits of the implanted lead before the IPG is placed.[7] Loss of efficacy after initial success was seen three times more frequently than in patients with the earlier non-tined leads as compared to tined leads.[11]

A prospective, European, multicenter study performed screening with tined lead in 94 patients with success demonstrated in 76.6%. At six months, follow-up data was available for 41 patients (20 with UI, 21 with NOUR). The patients with OAB had significant improvements in mean number of daily voids, incontinent episodes, and number of pads. Patients with NOUR had significant improvement in mean number of voids per day, self-catheterizations and catheterization volumes.[7] A single institution looked at quality of life (QOL) parameters after SNM with tined leads and found the majority (84%) was satisfied with the therapy and 80% would undergo the therapy again. Those patients who were satisfied were more likely to have had improved clinical response than those dissatisfied.[7] Finally, there are no long term studies looking at safety of tined lead use, but there does not appear to be significant concerns regarding infection or site discomfort.

## FLUOROSCOPY

The addition of fluoroscopy during percutaneous testing and lead positioning has markedly improved lead placement and subsequent clinical efficacy. SNM was initially employed using a screening test consisting of a bilateral percutaneous nerve evaluation (PNE). The PNE was performed without fluoroscopy and the temporary electrode was passed “blindly” through the S3 foramen using only anatomic/bony landmarks and motor responses to ensure proper placement. Spinelli *et al*. in 2003 first described the tined lead staged trial.[12] This technique uses intra-operative fluoroscopy to improve the accuracy of lead placement, and the self-anchoring tined lead device to minimize migration during the first stage. During previous traditional PNE testing neither fluoroscopy nor tined leads were used. A simple wire is placed percutaneously and secured through adhesive tape as it exits the skin. False negative screening tests occurred due to dislodged leads or improper initial placement. In the United States, experienced implanters shifted from using the PNE to routine use of the unilateral tined lead as an initial staged trial. This has significantly increased the percentage of patients undergoing screening who are ultimately chosen for permanent implant of the entire device.[7,13]

A resurgence of the bilateral percutaneous nerve evaluation (PNE) has occurred now that fluoroscopy is readily available in the office setting. Anterior-posterior (A-P) and lateral fluoroscopy are routinely used during PNE procedure to improve lead placement within the foramen. Two leads are placed to maximize possible therapeutic benefit. Patient interest and acceptance of this therapy may be increased by the fact that the test phase can be performed in the office under local anesthesia, and leads are easily removed at the end of the trial. This still leaves the possibility of a staged lead for those with a sub-optimal response, but also offers the advantage of implantation of the entire device (tined lead and IPG) in a single operative procedure for those who have a successful office screening PNE. While there remains a chance that the permanent implant may not be as successful as the PNE, the risk of this is very low with experienced InterStim implanters. On the other hand, a staged tined lead implant does not preclude the potential need for a future revision due to sub-optimal efficacy over time. The selection of office PNE versus permanent tined lead placement as an initial screening method should be tailored to the particular needs and presenting symptoms of the patient.

## IMPLANTABLE PULSE GENERATOR

The original IPG is larger and has an average battery life of 7-10 years. It is preferred in patients who require high voltage for stimulation in order to maximize the time to IPG replacement. The InterStim II device, introduced in 2006, is 50% lighter and smaller. The smaller generator allows for a smaller incision and pocket to be created leading to less discomfort and higher patient acceptance.[7] It is ideal in thin patients, pediatric patients and those with lower stimulation intensity requirements determined at time of test stimulation. Due to the smaller size, the average battery life is only three to five years. Care in placing the lead close and parallel to the nerve so that required stimulation thresholds are as low as possible on all four sites is key in optimizing battery life for either device.[1] The newer system also has upgraded software providing additional patient programming and data tracking opportunities.[9]

## EXPANDING INDICATIONS

Interstitial cystitis (IC) is a condition characterized by symptoms of urinary frequency, urgency and pelvic pain. Pharmacotherapy is the current mainstay of treatment, but is inadequate for many. While the urinary symptoms are approved indications for neuromodulation, pain is not. In a study of 21 refractory IC patients treated with SNM, there were improved subjective pain reports and decreased narcotic usage at a mean of 15.4 months follow-up.[14]

Chronic pelvic pain in the absence of urinary symptoms is also a difficult problem to treat. SNM was evaluated in one series of ten patients with chronic, refractory pelvic pain without significant voiding symptoms. Improvement of at least 40% after PNE qualified patients for permanent implant. Median follow-up was 19 months (6–74 months) after implantation. Six of 10 patients experienced substantial benefit. SNM decreased subjective pain severity and number of hours with pain.[15]

Neurogenic voiding dysfunction can lead to serious consequences including UTIs, stones, incontinence, and obstructive uropathy. A small series of spinal cord injury patients underwent sacral anterior root stimulation. Incontinence was improved by 73%, but many also had improvements in defecation and UTI occurrence.[14]

Fecal incontinence is currently treated with medications, pelvic floor biofeedback and surgery. The role of SNM has been explored by Altomare *et al*. in a multicenter center study of 94 fecal incontinent patients. 63% of these patients (60) went on to permanent lead placement and 87% (52) had long term follow-up--mean 74 months. 75% maintained at least 50% improvement after more than five years. They showed significant improvements in physical, social and emotional functioning on QOL domains. Manometric data for resting and squeeze anal pressure significantly improved. Urgency sensation and maximal volume tolerated showed a trend towards reduction.[16]

Dysfunctional elimination syndrome in children includes disturbances of the GI and urinary tract. Reinberg and colleagues looked at 20 patients’ refractory to medical therapy. SNM showed resolution or improvements in urinary incontinence (88%), frequency and urgency (89%), nocturnal enuresis (69%) and constipation (71%) with a median 27 months follow-up.[17]

## PUDENDAL NERVE STIMULATION

The pudendal nerve as the site of stimulation during neuromodulation has been an area of continued interest over the years. The pudendal nerve originates from S2, S3, and S4 sacral nerve roots. Direct pudendal nerve neuromodulation provides broader afferent stimulation vs. conventional S3 selection. Pudendal nerve stimulation (PNS) has been shown to inhibit the micturition reflex, increase bladder capacity and quiets uninhibited detrusor contractions. A prospective, single-blinded, randomized, crossover trial was performed by Peters *et al*. comparing sacral nerve stimulation (SNS) and PNS for voiding dysfunction. Thirty patients had a sacral tined lead placed, with a second pudendal tined lead placed on the same side. Following a seven-day trial, 80% of the patients experienced a favorable response with subsequent IPG placement. Significant improvements in symptoms were seen with both PNS and SNS including urgency, frequency, bowel function and pelvic pain. While objective changes in voiding symptoms were not statistically different, a majority of the subjects found greater subjective symptom improvement and comfort with the pudendal lead (79%). A drawback of this study is that EMG monitoring was performed with pudendal lead placement, but not the sacral lead. The more precise placement of the pudendal lead could have resulted in lower stimulation thresholds, and less collateral stimulation. These differences may account for the apparent subjective superiority of the more carefully placed leads.[18]

## COST CONSIDERATIONS

The total costs of OAB to society are significant. In the United States an estimated 12.6 billion dollars per year is spent on OAB.[19] Approximately 16-45% of adults have OAB with or without urinary incontinence.[20] One study sought to estimate the health care utilization costs before and after of SNM implant in 65 patients.[21] Utilization costs for the year before and the year after implantation were derived from Medicare CPT coding and reimbursement data. Specifically hospital and clinic visits, diagnostic and therapeutic procedures, and prescriptions Drug costs came from the actual pharmacy costs. Outpatient visits decreased by a mean of 2.2 (*P* < 0.0001) for urinary symptoms in the 12 months after implantation. The average yearly office visit expenses were reduced by 73% or from $994 to $265 per patient. Costs of diagnostic and therapeutic procedures performed decreased by 0.97 (*P* < 0.0001) after implant, which means a decrease from $733 to $59 per patient (*P* < 0.0001). Drug costs were significantly decreased (*P* < 0.02) from $693 to $483 per patient. The overall cost savings was a 30% reduction in drug expenditures and a 92% reduction in outpatient doctor visits and diagnostic and procedure costs. Based on this information, when a patient obtains clinical benefit from SNM health care expenditure stands to see a substantial decrease. This may be another argument for offering SNM earlier in the treatment algorithm.

## ALTERNATIVE THERAPY

Botulinum toxin (BTX) has been used urologically for OAB due to neurologic and non-neurologic disorders. BTX works by inhibiting acetylcholine vesicle formation and therefore its release at the neuromuscular junction. This leads to paralysis of the affected muscle group.[22]

BTX is considered by many as an alternative to SNM for refractory OAB. Several studies have demonstrated the effectiveness of intravesical BTX as a treatment for OAB. Duthie and colleagues reviewed randomized and quasi-randomized controlled trials of BTX and found outcomes similar to anticholinergic therapy reviews.[23] Improvements in incontinence episodes, quality of life, maximum detrusor pressure and bladder capacity were reported for BTX vs. placebo. A randomized, double-blind, placebo-controlled trial from the United Kingdom (UK) was recently published. Participants with idiopathic detrusor overactivity were randomized to receive either 200 U of BTX-A or placebo At 12 weeks, patients were ‘unblinded’ and an open-label follow-up in the BTX-A group occurred at 24 weeks. Overall QoL was significantly improved in the BTX-A treated patients compared with placebo in the blinded part of the study and this benefit persisted for at least 24 weeks.[24]

Since the effects of intravesical BTX generally last approximately six to nine months, patients require repeat injections to maintain a benefit. The cost of repeated injections can add up over time [Table 3]. The need to perform clean intermittent catheterization for urinary retention is a known side effect of BTX bladder injections in some individuals. The risk of retention and need for multiple procedures, sometimes twice per year, causes us to consider BTX injections usually after a trial of SNM. While the long term risks of BTX are unknown, five year data is available for SNM. SNM also has a potential additional benefit on pelvic pain and bowel symptoms, which cannot be anticipated with BTX. Also, no standards exist as to the dose or concentration of BTX to use or the number of sites to inject. While BTX therapy has promise it is still evolving and requires more research.

**Table 3:** Charged amounts for BTX vs. SNM

3 BTX claims reviewed - Average billed charge = $9,211 - Used CPT code 53899 - Charge included facility, drug, anesthesia, surgical 10 SNM claims reviewed - Average billed charge = $40,655 - Used CPT 64581 - Charge included 1° and 2° procedures, facility, implants, anesthesia, surgical

Currently, in the US, BTX use for OAB is considered investigational and an “off label” treatment for OAB. Because the medication is not FDA approved for the treatment of OAB many insurances do not cover this treatment for patients. Cost of BTX-A for the treatment of OAB has been addressed in the (UK). The conclusion of the study was that BTX-A was “highly likely to be cost-effective” in the treatment of OAB secondary to neurologic and non-neurologic causes when compared to standard OAB care in the UK.[25] Thus far, intravesical BTX injection for refractory OAB appears to be a safe and effective treatment without significant adverse effects.[26]

## FUTURE MODIFICATIONS

Future developments will likely include rechargeable batteries with longer total functional life. Modernized screening tools along with improved patient and clinician interfaces are almost a certainty. Development of MRI compatible systems and improved lead guidance for pudendal placements, as well as alternate stimulation targets (dorsal genital nerve) may also be in store.

## SUMMARY

Many patients with intractable non-neurogenic OAB symptoms have been successfully treated with SNM, and this therapy has thus emerged as a standard of care in the United States and Europe for this patient population.[2] The therapy has also been successful for some patients with idiopathic NOUR. The long-term effectiveness of SNM for these indications is well documented. There is increasing evidence that the therapy is also efficacious for patients with pelvic pain syndromes, fecal incontinence, neurogenic voiding dysfunction, and dysfunctional elimination syndrome. Routine use of fluoroscopy is essential for identifying the level and course of the appropriate sacral nerve root. Use of the permanent tined lead, along with local anesthesia and IV sedation, have improved lead implantation by allowing for sensory feedback during lead placement. The combined techniques allow for screening to be moved to the office setting making this therapy more widely accepted among patients. Smaller, lighter batteries have improved cosmesis and expanded use of this therapy to children. Future advancements will likely expand the indications, ease of use, and efficacy in the treatment of many patients with challenging pelvic disorders.
